# Are People Worse Off in a Mental Health Treatment Paradigm Where Medication Is Deemphasised? A Naturalistic Noninferiority Trial of an Initiative to Improve Patient Choice

**DOI:** 10.1177/00207640251390930

**Published:** 2025-11-23

**Authors:** Kari Standal, Ole André Solbakken, Jūratė Šaltytė Benth, Allan Abbass, Kristin S. Heiervang

**Affiliations:** 1 District Psychiatric Center Nedre Romerike, Akershus University Hospital, Lørenskog, Norway; 2 Department of Psychology, University of Oslo, Norway; 3 Institute of Clinical Medicine, Campus Ahus, University of Oslo, Norway; 4 Health Services Research Unit, Akershus University Hospital, Lørenskog, Norway; 5 Faculty of Medicine, Dalhousie University, Halifax, NS, Canada; 6 Research and Development Department, Division of Mental Health Services, Akershus University Hospital, Lørenskog, Norway; 7 Centre of Medical Ethics, Faculty of Medicine, University of Oslo, Norway

**Keywords:** medication-free, mental health care, psychotropics, outcome, choice

## Abstract

**Background:**

Norwegian health authorities have established dedicated units for medication-free mental health treatment (MFT) to enhance patient choice. These services place greater emphasis on psychosocial and psychotherapeutic interventions than traditional care. They aim to be free from coercion and pressure regarding medication, rather than totally absent of medication.

**Aims:**

This study evaluates whether outcomes for patients receiving MFT are noninferior to those receiving treatment as usual (TAU).

**Method:**

A noninferiority analysis was conducted using the Outcome Questionnaire-45.2 (OQ-45.2) to assess changes from admission to discharge. Two datasets were analysed: a smaller research sample with repeated measures and two comparison units (Sample R; n = 59 + 124), and a larger quality register sample with single-measure data and one comparison unit (Sample Q; *n* = 140 + 238). In sample R, associations between clinical and demographic variables and treatment outcomes were also explored.

**Results:**

Participants in both treatment conditions showed substantial improvement. In Sample R, changes between groups were not statistically significant, and the noninferiority analysis was inconclusive. In Sample Q, intention-to-treat analyses indicated superiority for MFT, while sensitivity analyses excluding dropouts supported noninferiority.

**Conclusions:**

Findings suggest that MFT is not associated with inferior short-term treatment outcomes in the population currently receiving this care. These results may reassure clinicians and policymakers in supporting patient choice and may assist patients in selecting their preferred treatment approach.

This study is registered at ClinicalTrials.gov (NCT03499080), date 17/04/2018.

## Introduction

Since 2015, mental health care in Norway has included services dedicated to medication-free treatment (MFT) of mental disorders. A nationwide governmental mandate to provide such specialist services is unprecedented globally.

The official aim of introducing MFT is to offer patients the opportunity to choose medication-free treatment, provided it is clinically justifiable (Meland & Dammen, 2015). A coalition of user organisations advocating for MFT described this initiative as promoting freedom from coercion, pressure, or persuasion regarding medication (Fellesaksjonen for medisinfri behandling, 2013, February 11). However, regional health authority protocols clarify that patients in “medication-free” units may still use psychotropic medication if they choose (Helse Nord, 2016; Helse Sør-Øst, 2016). Consequently, some have argued that the term “medication-free” is misleading (Reitan et al., 2024; Standal et al., 2024), although it remains the official designation.

In this article, MFT refers to services developed in response to the governmental directive, interpreted as being free from medication-related pressure and coercion, rather than entirely free of psychotropic medication. This pragmatic mandate necessitated treating the concept as a *sensitising* concept (Blumer, 1954), a loosely defined idea that is refined throughout the research process (Blaikie, 2010).

While MFT services are not uniform, the majority share common characteristics: inpatient open wards, prioritising individuals with severe mental disorders, and being influenced by a recovery-oriented tradition (Standal & Heiervang, 2018). The unit studied in this article differed from traditional treatment by using less medication, placing greater emphasis on alternative therapeutic approaches, allowing patients more autonomy in reducing or avoiding medication, restricting the use of controlled substances more, and expecting greater patient engagement in activity and responsibility (Standal et al., 2024).

Medication has been central to the treatment of severe mental disorders in Western medicine since the 1950s (Sohler et al., 2016). However, only about half of patients with severe psychiatric disorders adhere to their medication regimens (Semahegn et al., 2020). Recently, the World Health Organization (WHO, 2021) has criticised mental health care for being too restricted to a biomedical model of illness that centres on psychotropic medication. Alongside the United Nations (UN), the WHO has called for the elimination of practices that restrict individuals’ legal capacity, such as involuntary admission and treatment (United Nations [UN], 2006; WHO, 2021).

The deemphasis of medication has been particularly controversial in the treatment of psychosis and severe affective disorders. Consequently, these diagnostic groups are prioritised in the unit under study. Nonetheless, previous research indicates that MFT services attract a broader range of diagnostic categories. This may be due to limited access to psychosocial treatment options, negative experiences with medication, and individual values, attitudes, and beliefs (Standal et al., 2021).

Few studies have rigorously examined alternatives to medication for psychosis and bipolar disorder. Some reviews suggest that, for individuals with psychosis or acute illness, outcomes from paradigms that deemphasise medication may be comparable to those of standard treatment (Cooper et al., 2020; Lichtenberg, 2011). Cooper et al. (2020), for example, conducted a systematic review of psychosocial interventions for individuals with schizophrenia spectrum disorders who were on minimal or no antipsychotic medication. Eligible studies included empirical investigations of psychosocial interventions for individuals diagnosed with schizophrenia, schizoaffective disorder, delusional disorder, non-affective psychosis, or psychotic disorder, who were either not taking antipsychotics (including placebo) or were following an antipsychotic minimisation strategy (e.g. intermittent treatment, where medication is used only during symptomatic periods, or postponement, where antipsychotics are withheld for the first 2–6 weeks). The review identified nine interventions tested across 17 studies (*N* = 2,250), including eight randomised controlled trials. Most studies reported outcomes for the intervention which were comparable to those of the control group. However, the quality of the studies was problematic. Lichtenberg (2011) reviewed community-based residential homes designed to treat individuals with acute psychiatric illness, aiming to replace—not merely delay—acute hospitalisation. These settings typically featured fewer residents than hospital wards, a supportive and caring milieu with frequent therapeutic contact with staff, a deemphasis on medication, and efforts to destigmatise psychosis. The models varied in their use of psychotropic medication, focus on first-time admissions, and staff composition. Lichtenberg concluded that these alternative models were comparable to standard inpatient care in terms of clinical and psychosocial outcomes and were generally less costly. However, the findings should be considered preliminary, as significant limitations remain in the evidence base supporting therapeutic community residential alternatives to standard inpatient care. Further research is needed to address questions of cost-effectiveness, therapeutic efficacy, and long-term outcomes.

Established pharmacological treatments have demonstrated short-term symptom reduction for psychosis, bipolar disorder, and depression when compared with placebo (Chen & Shan, 2019; Chien & Yip, 2013; Cipriani et al., 2018; Gartlehner et al., 2017; Hasan et al., 2020; Helsedirektoratet, 2013; Hengartner, 2020; Jakobsen et al., 2017, 2020; Khan et al., 2018; Leichsenring et al., 2022; Leucht et al., 2017; Levenberg & Cordner, 2022; Luoma et al., 2020). Nonetheless, debates persist regarding the clinical significance of antidepressant effects (Jakobsen et al., 2020; Pies, 2012; Yapko, 2013) the strength of the evidence base for bipolar disorder treatments (Butler et al., 2018; Helsedirektoratet, 2012; Kadakia et al., 2021; Morsel et al., 2018; Pfennig et al., 2013) and the justification for long-term pharmacological use (Alvarez-Jimenez et al., 2016; Baghai et al., 2012; Correll et al., 2018, 2022; Goff et al., 2017; Hengartner, 2020; Moncrieff et al., 2020; Ralph & Espinet, 2018; Smedslund et al., 2018; Suvisaari et al., 2018). Additionally, pharmacotherapy is associated with a range of adverse effects (Abdullah et al., 2019; Bai et al., 2020; Ceraso et al., 2020; Chen & Shan, 2019; Chien & Yip, 2013; Correll et al., 2018; Davies & Read, 2019; Farah et al., 2016; Hengartner, 2020; Horowitz et al., 2021; Jakobsen et al., 2020; Levenberg & Cordner, 2022; Moncrieff et al., 2020; Sinyor et al., 2020; Yapko, 2013) that must be weighed against their benefits.

In Norway, concerns have been raised about the potential health consequences for individuals who choose MFT (Røssberg, 2016). Nyttingnes and Rugkåsa (2021) summarised the main stakeholder concerns as follows: medication coercion rates (primarily raised by the Ministry of Health), patient autonomy (emphasised by supporters), and the need for evidence-based treatment (highlighted by critics). The Norwegian Ministry of Health has argued that MFT is important for reducing coercion and addressing patients’ negative experiences with mental health services. The user coalition (Fellesaksjonen for medisinfri behandling) and other MFT proponents share this perspective, although their critique of existing care practices has been more pointed. They argue that separate units are necessary to ensure genuine treatment choice and to avoid coercive environments. Furthermore, they maintain that the MFT label is essential to enable reluctant patients to access care. In contrast, critics of MFT contend that there is insufficient evidence to support the model, particularly in comparison to the robust evidence base for the efficacy of antipsychotic medication. On the one hand, treatment without scientific evidence is ethically dubious and reluctance to receive medication can be driven by lack of insight. On the other hand, the critics consider that the current services provide room for patients’ choice and treatment of nonresponders. However, they oppose the promotion of MFT as a distinct healthcare service, arguing that it risks creating an artificial divide between biological and psychosocial treatments and may contribute to the stigmatisation of medication use (Nyttingnes & Rugkåsa, 2021).

Our study is the first to compare MFT, as implemented in this form, with standard treatment. We evaluated whether MFT was noninferior to treatment as usual (TAU) under naturalistic conditions, using the Outcome Questionnaire 45-2 (OQ-45.2) as the primary measure of mental health outcomes. Additionally, we explored how various treatment-related and patient-related factors were associated with outcomes, including subgroup analyses by diagnosis. We also examined associations with common demographic variables such as age and gender. The impact of treatment duration was assessed as this was known to differ between treatment conditions and may represent an important aspect of the overall picture. Associations with the dosage of primary medication groups and total dose changes during treatment were examined, given that the deemphasis on medication is central to the MFT model. We also explored associations with the presence of psychosis or bipolar disorder diagnoses, as these groups are prioritised in the medication-free unit due to the traditionally strong emphasis on pharmacological treatment. For the same reason, outcomes for the subgroup with either psychosis or bipolar disorder were analysed separately.

## Method

### Setting

The study was conducted at one MFT inpatient unit and two TAU inpatient units within a general university hospital serving a catchment area of approximately 500,000 people, encompassing both urban and rural communities. The MFT unit is representative of such services in terms of its most common characteristics: it is an open inpatient ward, prioritises individuals with severe mental disorders, and is influenced by a *recovery*-oriented approach (Standal & Heiervang, 2018). The characteristics of the included treatment units are presented in Table 1.

**Table 1.** Characteristics of the Included Treatment Units.

Features	MFT unit	Comparison unit 1	Comparison unit 2
Target population	People over age 18 years in need of voluntary inpatient mental health care. Exclusion: active addiction, acute suicidal behaviour, or acute aggressive/violent behaviour		
	People with psychosis or bipolar disorder wanting MFT are prioritised	Patients needing transfer from the acute ward are prioritised	
Treatment programme	Recovery oriented (Slade et al., 2012). Elements from the traditions of the affect consciousness model (Monsen & Monsen, 2000), a feedback-informed framework (Miller et al., 2015), open dialogue (Seikkula & Arnkil, 2013), and techniques from basal exposure therapy (Hammer et al., 2018).		Cognitive milieu therapy, network meetings, counselling, and diverse group activities
Weekend-policy	5-day unit where patients go home for the weekends	7-day unit, but the main rule is patients go home in the weekend.	7-day unit
Treatment duration	Typically 8 weeks	Varied, mean 4 weeks	Varied, typically 6–8 weeks
Ordinary treatment places	7	9	14

### Design

This study was part of a larger research project registered at ClinicalTrials.gov (Identifier: NCT03499080). The design was observational, conducted within a naturalistic treatment setting. We compared clinical mental health outcomes between a MFT unit and TAU units. Data were collected using questionnaires completed by both patients and clinicians. Changes in the primary outcome measure from baseline to the end of treatment were compared between the MFT and TAU conditions.

An observational design was necessary because patient choice is a fundamental principle of MFT services. Random assignment to MFT or TAU was not feasible, as it would have conflicted with the ethical imperative to respect patient autonomy. Nevertheless, it remains important to understand the consequences of offering treatment alternatives and allowing patients to choose, particularly considering concerns about treatment adherence and the potential need for coercive interventions.

### Inclusion and Sampling

Table 2 presents the inclusion and sampling details for the two data sources. Responses to the primary outcome measure were obtained from two overlapping samples: participants in the research project (Sample R) and a local quality register covering two units (Sample Q). According to Norwegian law on patient journal § 6 (Lov om behandling av helseopplysninger ved ytelse av helsehjelp [Law on patient journal], 2015) (2015) has not been listed in the references. Please check.] and law on health personnel § 26 (Lov om helsepersonell m.v. [Law on health personell a.o.], 2000), quality improvement projects are exempt from the requirement for informed consent although results may be published in anonymised form. The research study (sample R) is approved by the Regional Committee for Ethics in Research (REK) (2017/1056/REK Sør-Øst B.), as well as the Privacy Ombudsman at Akershus University Hospital (17-134). The quality register (Sample Q) was approved by the Privacy Ombudsman at Akershus University Hospital (25-2018), including approval for publication of anonymous results.

**Table 2.** Inclusion and Sampling.

Data set	Sample R	Sample Q
Units included	All three	MFT and comparison unit 1
Consent required	Yes	No
Other inclusion criteria	Patients on a planned stay during the recruitment period capable of completing forms in Norwegian. The exclusion criteria were emergency stays, self-referral admissions, or inability to participate (being unable to complete forms in Norwegian).	
Inclusion periods	May 2018 to April 2020, and September 2020 to March 2021, with an intermittent break due to the COVID-19 pandemic. Only one of the two TAU continued recruiting patients after the break.	June 2017 to May 2022, but we excluded the data from the medication-free unit in period April-June 2021, due to a temporary shift to outpatient treatment caused by the pandemic
Sampling procedures	All eligible patients were asked for consent by treatment staff upon admission.	OQ-45-2 responses were gathered as part of the treatment.
Sample size	183	378
Response rate	46.2%	91.9%
Measures included	OQ-45-2, covariates, descriptive sample measures	Only OQ-45-2

*Note*. Response rate = % included in study among all admitted in the period.

Self-referral admissions: A special arrangement made with certain patients wherein they can self-refer for short stays of a few days when needed. These do not follow the standard treatment programme.

### Sample Size, Power, and Precision

We assumed a standard deviation (SD) of 15 points for the primary outcome variable (OQ-45.2 Total Distress), with a noninferiority margin of 5 points. This margin was chosen to correspond to a Cohen’s *d* of approximately 0.3 in a normal population (Amble et al., 2014), representing a small to moderate effect size, consistent with previous studies (Connolly Gibbons et al., 2016; Driessen et al., 2017). Assuming a power of .80 and a one-sided significance level of .05, the required sample size was 224. Power calculations were conducted according to the principles described by Julious (2004) using the Sealed Envelope power calculator for continuous outcome noninferiority trials.

The required sample size was not achieved in sample *R* but was met in sample *Q*. Recruitment for sample *R* was more affected by the COVID-19 pandemic, as it required greater resource allocation. According to post hoc sensitivity analyses conducted using G*Power (Faul et al., 2009) sample *R* had sufficient power to detect medium-sized effects (Cohen’s *d* = .53).

### Measurements

Translated versions of clinical measures that are not standardised instruments are available in the Online Resource. Permissions were obtained where required.

#### Outcome Questionnaire-45.2 (OQ-45.2)

The OQ-45.2 was developed to monitor outpatient progress on a weekly basis (Lambert et al., 2001) and is one of the most widely used patient-reported outcome measures (PROMs) for assessing mental health and psychosocial functioning (Gelkopf et al., 2022). It evaluates three core domains: symptom distress, interpersonal functioning, and satisfaction with social role performance—areas considered essential for assessing patient improvement (Lambert et al., 2001). The OQ-45.2 has also been validated in inpatient settings, demonstrating adequate psychometric properties and high sensitivity to change (Doerfler et al., 2002). It was selected as the primary outcome measure due to its suitability for patients with a wide range of diagnoses, sensitivity to change over short periods, and that it is considered brief and easy to administer (Lambert et al., 2001). It has demonstrated good psychometric properties, including in Norwegian settings (Amble et al., 2014; Lambert et al., 1996).

- Range: 0–180 (Higher scores indicate greater symptom severity)
- Timing: Administered at Baseline, weekly during treatment, and at end of treatment
- Source: Patient-reported

Tables 3 and 4 present the covariates and descriptive sample measures

**Table 3.** Measures of Covariates.

Measure	Subgroup		Timing	Source
Dose change all psychotropics		Psychotropic drugs were grouped according to the Norwegian medication handbook (Foreningen for utgivelse av Norsk legemiddelhåndbok [Society for publication of Norwegian Medication Handbook], n.d.). Daily doses were converted to number of Defined daily doses (DDD) according to WHO (n.d.)	B, E	C
Dose at treatment end	Anxiolytics/ hypnotics		E	
	Antidepressants			
	Medication for hyperkinetic disorders and narcolepsy			
	Antipsychotics			
	Mood stabilisers			
Duration of treatment (weeks)				
Age			B	P
Gender				
Psychosis diagnosis (F20-29)		ICD-10, grouped as shown in Supplemental Table S2	E	C
Bipolar diagnosis (F30-31)				

*Note*. B = baseline; E = end of treatment; P = patient; C = clinician.

**Table 4.** Measures of Descriptive Sample Characteristics.

Measure	Description	Timing	Source
Main diagnosis	ICD-10, grouped as shown in Supplemental Table S2	E	C
Use of psychotropics	A combined variable was made most consistent with available sources.	B, E	P, C
Affect Integration Inventory Short Form-42 (AII-42)	A 42-item short version of the AII, a medium-length (112 items) self-rated assessment instrument that endeavours to measure capacities for experience and expression of nine affect states. These are important parts of the construct affect integration, the capacity to utilise affects for personal adjustment. A recent study has found satisfactory reliability, sound internal structure, and associations with external criteria, indicating good convergent and discriminant validity (Solbakken et al., 2017). Range 0→9	B	P
Global assessment of functioning (GAF)	The GAF scale is one of the axes in the *Diagnostic and Statistical Manual of Mental Disorders* (DSM) from DSM-III-R (Karterud et al., 1998) until DSM-5. The multiaxial system was discarded in DSM-5 (Kress et al., 2014). From 1998 to 2020, Norwegian health authorities recommended all health institutions to use a minimum set of basic data, which included a split version of GAF called S-GAF (Karterud et al., 1998; Lie, 2019). Patient scores are ranged on two scales from 0 to 100 regarding their symptoms and functioning. While the psychometric properties of GAF are disputed (Kress et al., 2014; Lie, 2019), the measure is short, widely applied, and was mandatory in hospitals at the start of the recruitment period.		C

*Note*. B = baseline; E = end of treatment; P = patient; C = clinician.

### Response Rates and Representativeness

To assess response rates (i.e. the percentage of individuals included in the study among all admissions during the recruitment period) and the representativeness of Sample R, we accessed anonymised statistics from electronic patient records for the relevant period. The total sample size included admissions to the units, excluding emergency and self-referral admissions. Due to differences in data sources for self-referrals, the sample sizes reported in the flow chart and the Online Resource differ slightly, with the flow chart more closely reflecting our inclusion criteria. The Online Resource (Supplemental Tables S1–S2), which includes age, gender, and diagnosis, covers all admissions during the recruitment period, excluding emergency admissions and readmissions within 30 days, to allow for better comparison with Sample R.

To estimate the response rate for Sample Q, the total number of admissions during the recruitment period was approximated based on the mean weekly admissions observed during Sample R recruitment. Due to an organisational change during the recruitment period that affected admission numbers in TAU units, the mean for Period 1 was used to extrapolate admissions prior to 2020, and the mean for Period 2 was used for admissions after 2020. For the MFT unit, which had fewer admissions and no known historical changes, the overall mean across both periods was considered the most reliable estimate. As the register is anonymous, there is some uncertainty regarding whether all planned admission types were included; however, this approach provided the most reliable estimate available.

### Psychometrics

Reliability was excellent for all multi-item scales in both samples, with Cronbach’s alpha values exceeding .90 (see Supplemental Table S3, Online Resource).

### Analyses

Missing items in the OQ-45.2 were handled using the expectation–maximisation method in Sample R and according to clinical scoring guidelines in Sample Q. At baseline and treatment termination, missing data appeared to be missing at random, though this was not the case for intermittent time points (see Supplemental Table S4, Online Resource).

Participant characteristics were summarised using means and standard deviations (SDs) or frequencies and percentages. Group comparisons were conducted using independent-samples *t*-tests or χ²-tests, as appropriate.

The primary outcome, OQ-45.2, was administered repeatedly at baseline, during treatment, and at discharge. The primary noninferiority analysis compared changes from baseline to discharge between groups using a one-sided 95% confidence interval (CI) from independent-samples *t*-tests, tested against a noninferiority margin of –5 points. Identical analyses were performed on both datasets. In Sample R, missing values were imputed using session-by-session OQ-45.2 data. Predicted discharge scores were generated within each group using linear mixed models with random intercepts for patients and fixed effects for treatment weeks. In Sample Q, no information was available on treatment duration or a designated endpoint measure; therefore, the last available score—typically collected 1 week prior to discharge—was used as the endpoint. For cases with only one assessment, the last observation was carried forward. As a sensitivity analysis, the noninferiority test for Sample Q was repeated after excluding cases with only one assessment, as these were unevenly distributed across conditions (92 in TAU vs. 4 in MFT). In contrast, Sample R included only 8 such cases in total.

In line with a naturalistic approach, we aimed to study the phenomenon “as is,” reflecting the population receiving the different treatments in real-world conditions, with minimal assumptions. Accordingly, we did not control for covariates in the primary noninferiority analysis but explored their influence in secondary analyses.

Multiple linear regression models were used to examine associations between outcomes and pre-specified covariates: medication use, treatment duration, age, gender, and diagnoses of psychosis or bipolar disorder (Sample R). Outcomes for participants with either a psychosis or bipolar diagnosis were compared between treatment conditions using independent-samples *t*-tests. Multicollinearity was assessed through correlation analysis, and standard residual diagnostics were performed. All regression models included only participants with complete data on the relevant covariates.

The statistical analyses were conducted using IBM SPSS Statistics for Windows (Version 26; IBM SPSS, Armonk, NY, USA) and STATA (Version 17; StataCorp, College Station, TX, USA). Analyses were based on the intention-to-treat principle, except for the additional sensitivity analysis in Sample Q described above. The significance level for superiority analyses of primary and secondary outcomes was set at 0.05.

## Results

### Participant Flowchart

**Figure 1.**
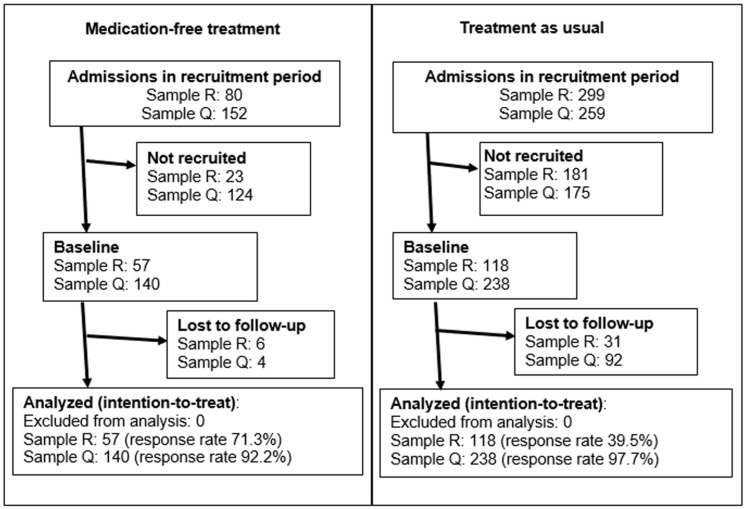
Participant flowchart based on responses to the main outcome questionnaire OQ-45.2. *Note*. Sample R = sample from research study; Sample Q = Sample from quality register.

Figure 1 shows the participant flowchart.

### Sample Characteristics

General sample statistics are shown in Table 5 and use of psychotropics in Table 6. Details in Online Resource.

**Table 5.** Descriptive Sample Characteristics.

Variables				*n* (each group)	Statistics	MFT	TAU	Difference between regimens	
								*p*-Value	Test
Gender			Female	183 (59 + 124)	*n* (valid %)	42 (71.2)	72 (58.1)	.087	χ^2^-test
			Male			17 (28.8)	52 (41.9)		
Age				183 (59 + 124)	*M* (*SD*)	38.6 (13.1)	43.7 (12.9)	.**013**	t-test
Treatment duration (weeks)				182 (59 + 123)		8.9 (2.2)	4.7(2.2)	**<.001**	
Diagnosis		Psychosis		182 (59 + 123)	*n* (valid %)	9 (15.3)	12 (9.8)	.277	χ^2^-test
		Bipolar				8 (13.6)	20 (16.3)	.636	
Mental health at baseline	OQ-45-2 baseline	R^a^		175 (57 + 118)	*M* (*SD*)	99.3 (21.1)	96.1 (23.1)	.372	*t*-test
		Q^b^		282 (111 + 171)		99.1 (21.0)	95.3 (27.9)	.198	
	AII-42			173 (56 + 117)		4.1 (1.2)	4.5 (1.2)	.**045**	
	GAF-S			177 (58 + 119)		52.1 (8.6)	50.8 (6.3)	.252	
	GAF-F			177 (58 + 119)		49.8 (8.5)	50.9 (6.6)	.324	

*Note*. MFT = medication-free treatment; TAU = treatment as usual; t test = Independent samples t test; bold text = statistically significant.

a R = sample R (Research sample).

b Q = sample Q (Quality register).

**Table 6.** Use of Psychotropics.

Variables			*n* (each group)	Statistics	MFT	TAU	Difference between regimens	
							*p*-Value	test
Psychotropics use, baseline			183 (59 + 124)	*n* (valid %)	46 (78.0)	113 (91.1)	.**008**	χ^2-^test
Dose change			182 (59 + 123)	*M* (*SD*, IQR)	−0.4 (1.2, 1.1)	0 (1.2, 1.1)	.**040**	*t*-test
Dose at treatment end	Anxiolytics/hypnotics		182 (59 + 123)		0.4 (0.8, 0.6)	0.4 (0.7,0.7)	.947	
	Antidepressants	All	182 (59 + 123)		0.5 (0.8, 1.0)	0.9 (1.0, 1.5)	.**004**	
		Non-bipolar affective disorder	48 (11 + 37)		0.2 (0.4, 0.5)	1.2 (1.0, 1.0)	.**000**	
	HYP		182 (59 + 123)		0.1 (0.3, 0.0)	0.0 (0.2, 0.0)	.697	
	Antipsychotics	All	182 (59 + 123)		0.2 (0.4, 0.2)	0.5 (0.8, 0.8)	.**001**	
		Psychosis diagnosis	21 (9 + 12)		0.6 (0.6, 1.1)	1.9 (1.2, 1.5)	.**010**	
		Bipolar diagnosis	28 (8 + 20)		0.3 (0.6, 0.5)	0.9 (0.8, 1.3)	.114	
	Mood stabilisers	All	182 (59 + 123)		0.2 (0.4, 0.0)	0.3 (0.6, 0.0)	.183	
		Bipolar diagnosis	28 (8 + 20)		0.6 (0.7, 1.3)	1.1 (0.8, 1.3)	.163	

*Note*. MFT = medication-free treatment; TAU = treatment as usual; Dose = number of defined daily doses (DDD) according to WHO; t test = independent samples t test; HYP = medication for hyperkinetic disorders/narcolepsy; bold text = statistically significant.

The sample was generally representative when compared with other sources on admissions during the recruitment period on patient characteristics (Online Resource, Supplemental Tables S1 and S2). However, patients in the TAU group with psychosis and personality disorders were underrepresented by 10.2% and 9.2%, respectively, compared with hospital statistics. Although individuals with psychosis or bipolar disorder are prioritised in MFT, in practice, both treatment regimens included a wide range of diagnoses (Online Resource, Supplemental Table S2).

Participants in the MFT group were slightly younger, fewer used psychotropic medication at baseline, and they scored marginally lower on the Affect Integration Inventory (AII-42) at baseline compared to the TAU group. Otherwise, baseline mental health status did not differ substantially between groups. MFT participants had treatment stays approximately twice as long, experienced a greater reduction in psychotropic medication dosage during treatment, and had lower doses of antidepressants and antipsychotics at discharge compared to TAU participants. Most participants had used at least one type of psychotropic medication for more than 6 weeks, and overall, MFT participants had a longer history of psychotropic use (Online Resource, Supplemental Tables S7 and S8).

### Analyses of Differences in Change Between Regimens

**Figure 2.**
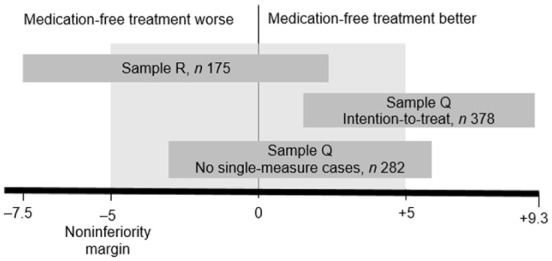
Graphic illustration of result of the noninferiority analyses of change on OQ-45.2 in medication-free treatment compared to treatment as usual. *Note*. Sample R = Sample from research study, Sample Q = Sample from quality register. Since participants with only one measure were unevenly distributed between conditions in sample Q, a separate analysis excluding these was performed on sample Q.

In the primary noninferiority analysis (Table 7), outcomes in sample R did not differ significantly between MFT and TAU. However, the lower bound of the one-sided 95% CI crossed the noninferiority margin of –5 points. Although not indicating inferiority, this prevented a definitive conclusion that MFT was noninferior to TAU in this sample. As the CI also included zero, the result was considered inconclusive (Piaggio et al., 2012). In the larger sample Q, the intention-to-treat analysis showed superiority for MFT. However, there was a disproportionate number of cases with only one measure in TAU. Sensitivity analyses excluding these cases demonstrated noninferiority for MFT. The results of the noninferiority analyses are summarised in Figure 2.

**Table 7.** Noninferiority Analysis of the Changes in the Primary Outcome (OQ-45-2) During Treatment.

Data set	MFT *n* *M* (*SD*)	TAU *n* *M* (*SD*)	Mean difference (95% CI)	Cohen’s *d* (95% CI)	*p* Value
Sample R, *n* = 175	57 14.0 (19.5)	118 16.7 (17.4)	–2.7 [–7.5, 2.2]	–0.2 [–0.4, 0.1]	.363
Sample Q, intention to treat, *n* = 378	140 12.0 (21.2)	238 6.7 (13.9)	5.3 [1.4, 9.3]	0.3 [0.1, 0.5]	.008
Sample Q, removed cases with a single measure, *n* = 282	136 12.4 (21.4)	146 10.9 (16.5)	1.5 [−3.0, 6.0]	0.1 [−0.2, 0.3]	.514

*Note*. MFT = medication-free treatment; TAU = treatment as usual.

### Impact of Covariates

Table 8 presents the results of a linear regression analysis examining the influence of covariates in sample R. In the multiple regression model, higher doses of antidepressants at the end of treatment were significantly associated with greater improvement on the OQ-45.2 (*p* = .037), as was a diagnosis of bipolar disorder (*p* = .012). Other variables—including age, gender, diagnosis of psychosis, treatment duration, change in overall medication dose, and dosages of anxiolytics/hypnotics, antipsychotics, mood stabilisers, or medications for hyperkinetic disorders/ADHD and narcolepsy—were not significantly associated with changes in outcome scores.

**Table 8.** Results of Linear Regression Analysis of Change in OQ-45.2 Scores.

Covariate		Multiple models	
		RC (95% CI)	*p* Value
Intercept		5.1 [–7.0, 17.2]	.405
Regimen (TAU – reference)		–7.7 [–15.7, 0.4]	.061
Number of defined daily doses (DDD)	Anxiolytics/hypnotics	0.7 [–3.2, 4.6]	.720
	Antidepressants	3.0 [0.2, 5.8]	.**037**
	Medication for hyperkinetic disorders and narcolepsy	–0.9 [–11.0, 9.2]	.861
	Antipsychotics	–4.3 [–9.0, 0.4]	.072
	Mood stabilisers	–3.8 [–10.1, 2.6]	.247
	Total change	2.1 [–0.1, 4.3]	.067
Age		0.1 [–0.2, 0.3]	.639
Gender, male		3.1 [–2.4, 8.6]	.272
Treatment duration		1.1 [–0.1, 2.4]	.063
Psychosis diagnosis		4.8 [–5.2, 14.9]	.342
Bipolar diagnosis		12.3 [2.8, 21.8]	.**012**

*Note*. MFT = medication-free treatment; TAU = treatment as usual; RC = regression coefficient; DDD = defined daily dose according to WHO, n = 174 (MFT 57, TAU 117); bold text = statistically significant.

A comparison of outcomes between treatment regimens for the patient subgroup diagnosed with bipolar disorder or psychosis showed no indication that this group fared worse in MFT compared to TAU (Supplemental Table S5, Online Resource).

### Outcome by Treatment Regimen

Treatment outcomes for each group are presented in Table 9. Patients in both regimens demonstrated substantial improvement over the course of treatment.

**Table 9.** Treatment Outcomes by Regimen (OQ-45.2).

Data set	Analysis	Statistics	MFT	TAU
Sample R	Intention to treat	Mean change	14.0	16.1
		(95% CI)	[8.8, 19.2]	[13.1, 19.2]
		*p*-Value	**<.001**	**<.001**
		Cohen’s *d*	0.7	1.0
		(95% CI)	[0.4, 1.0]	[0.8, 1.2]
		*n*	57	117
Sample Q	Intention to treat	Mean change	12.0	6.7
		(95% CI)	[8.5, 15.6]	[4.9, 8.4]
		*p*-Value	**<.001**	**<.001**
		Cohen’s *d*	0.6	0.5
		(95% CI)	[0.4, 0.7]	[0.3, 0.6]
		*n*	140	238
Sample Q	No single-measure cases	Mean change	12.4	10.9
		(95% CI)	[8.7, 16.0]	[8.2, 13.6]
		*p*-Value	**<.001**	**<.001**
		Cohen’s *d*	0.6	0.7
		(95% CI)	[0.4, 0.8]	[0.5, 0.8]
		*n*	136	146

*Note*. MFT = medication-free treatment; TAU = treatment as usual, Range of OQ-45-2 = 0←180. The differences were calculated as the baseline value minus the end of treatment value; a positive value implies a decrease in distress (i.e. improvement), and a negative value implies an increase within the group (i.e. deterioration); bold text = statistically significant..

## Discussion

Participants in both treatment regimens showed substantial improvement. The smaller dataset yielded an inconclusive noninferiority result, while the larger dataset indicated superiority for MFT in the full sample and noninferiority in sensitivity analyses. Across treatment conditions, higher antidepressant dosage and a diagnosis of bipolar disorder were associated with greater improvement.

Cases with only one assessment in sample Q may reflect very short treatment stays, which we know were more common in the TAU units. Alternatively, they may indicate higher dropout rates in completing outcome measures. Regardless of the cause, a single assessment precludes the detection of change, thereby introducing a potential bias against TAU in intention-to-treat analyses, as these cases generally showed improvement.

Taken together, the noninferiority analyses across both datasets suggest that any true difference in outcomes between MFT and TAU is likely negligible. When excluding the disproportionately distributed single-measurement cases, there is no indication that the larger dataset is more biased. Thus, it is reasonable to conclude that outcomes in MFT are likely noninferior to those in TAU.

Given the small sample sizes, the strength of associations with covariates should be interpreted with caution. While greater use of antidepressants was associated with larger short-term improvements, the effect size was small. This finding contrasts with results from another MFT unit, where patients who chose to discontinue medication experienced better outcomes. The use of medications for conditions most debated in the context of MFT—such as antipsychotics and mood stabilisers was not associated with mental health outcomes in our study. However, these relationships may be complex and difficult to detect. There were no indications that participants with psychosis or bipolar disorder fared worse in MFT compared to TAU, although subgroup sizes were small. Additionally, we found no evidence of significant withdrawal effects, as reductions in overall medication dosage were not associated with poorer outcomes. It is worth noting, however, that the overall reduction in dosage was modest and reflected a cautious tapering approach aimed at minimising adverse effects.

Treatment in the MFT unit involved more psychosocial interventions, including longer admissions. Therefore, similar outcomes should not be expected from simply removing medication without providing additional therapeutic support. The observed outcomes likely reflect the comprehensive therapeutic context of MFT (e.g. longer stays, structured support), rather than medication status alone. However, treatment duration itself was not significantly associated with outcomes. Longer admissions are more costly in the short term, although there will be reductions in costs associated with medications. The long-term cost-effectiveness of the MFT approach remains uncertain and warrants further investigation, including assessments of long-term outcomes and healthcare resource utilisation. In addition to monetary health-care costs, the costs of patients’ experiences with side effects and the negative effects of medications must also be considered. Cost considerations are also impacted by the view of mental illnesses. In the Norwegian debate, it has been pointed out that learning to cope with feelings has important value for individuals and society (Karterud, 2023). If one believes that feelings have a function and meaning, the way one deals with them has broader implications than merely alleviating symptoms. As stated by Karterud (2023), feelings are linked to norms and morals; consequently, a liberal democratic society necessitates citizens with high affect consciousness. In our study, patients’ experiences with medication included emotional flattening, feeling “zombielike” or “less human,” feeling empty and tired, having suicidal thoughts, and misusing the medicine. One can imagine that these negative experiences have great costs for both individuals and society. Taken together, the ultimate balance of costs and gains is uncertain and probably highly individual.

Our findings should be viewed considering the primary aim of MFT units and the motivations of patients who choose this form of care. The central goal of MFT is to safeguard individuals’ right to choose their treatment, rather than to maximise clinical outcomes. This approach recognises that mental health care encompasses more than pharmacological intervention, and that medication should not be a prerequisite for receiving support. In this context, shared decision-making about medication becomes a critical factor in evaluating the need for MFT services.

In addition to concerns about side effects, some patients in traditional care settings report feeling pressured to use medication. For these individuals, access to services that explicitly support non-medication-based approaches is essential (Standal et al., 2021). Coerced medication can be experienced as traumatic (Nyttingnes et al., 2016) and MFT units may attract individuals who would otherwise avoid treatment altogether. If MFT were associated with significantly poorer outcomes or serious deterioration, this would be important information for patients and providers when making treatment decisions. However, our findings do not support such concerns.

Our research contributes to the existing literature by providing further evidence that viable alternatives to TAU may exist, particularly regarding the role of medication in mental health care—a domain where evidence for the most severe disorders remains limited and contested (Cooper et al., 2020; Lichtenberg, 2011; Swartz et al., 2018).

To our knowledge, this is the first study to compare the Norwegian MFT initiative with TAU. Although the evidence base for non-medication alternatives in severe mental disorders is still scarce, current medication-focused paradigms also face challenges. For example, the long-term effects of psychotropic medications remain controversial, with mixed findings reported across studies (Alvarez-Jimenez et al., 2016; Arıkan et al., 2023; Baghai et al., 2012; Belge et al., 2023; Correll et al., 2018; Goff et al., 2017; Hengartner, 2020; Moncrieff et al., 2020; Smedslund et al., 2018; Suvisaari et al., 2018; Zipursky et al., 2020). Moreover, side effects are well-documented (Bai et al., 2020; Cai et al., 2023; Ceraso et al., 2020; Correll et al., 2018; Croatto et al., 2023; Davies & Read, 2019; Hengartner, 2020; Horowitz et al., 2021; Jakobsen et al., 2020; Levenberg & Cordner, 2022; Moncrieff et al., 2020; Sinyor et al., 2020) and represent important concerns for patients (Bjørgen et al., 2020; Øvernes, 2019). Additionally, severe mental disorders continue to impose a substantial global health burden despite current treatment efforts (GBD 2017 Disease and Injury Incidence and Prevalence Collaborators, 2018), underscoring the need for humility in determining the most appropriate approaches for individual cases and for embracing a broader range of treatment options.

### Strengths and Limitations

This exploratory study examined a typical MFT unit in a naturalistic setting, which was essential for evaluating this novel and under-researched treatment model. Due to the central importance of patient choice in MFT, randomisation to MFT or TAU was not feasible. This limitation reduced control over confounding variables and introduced uncertainty regarding causal inference. However, baseline mental health measures were similar across treatment conditions. We cannot assume equivalence between the samples, but we can describe the treatment trajectories of individuals who want a MFT treatment programme when they are able to choose this, compared to people choosing TAU. Even if they differ in important aspects, they are the ones the treatment service is intended for. Therefore, we believe these data are important.

The results of our study may have been influenced by the unique characteristics of the local MFT unit as well as the larger national context. Hence, we do not know the generalisability outside Norway, and further research in diverse systems might elucidate the generalisation of findings.

The statistical power in sample R was less than ideal due to unforeseen events during recruitment, while sample Q provided adequate power. To our knowledge, no other data are published on outcome of Norwegian MFT-clinics compared to TAU.

The practical requirement of being able to fill out forms in Norwegian may have excluded people newly arrived in Norway as well as the most acutely ill. The response rates were good for sample R and sample Q in MFT, while the response rate for TAU in sample R was somewhat lower, but the sample characteristics were similar to hospital statistics, supporting representability (Online resource, Supplemental Tables S1 and S2).

The relationships between conditions, medications, and outcomes may be complex, and we had insufficient statistical power to perform complex subgroup analyses, limiting the interpretation of the influence of the included covariates.

The unit under study, like most MFT units in Norway, is at an intermediate level of care and excludes the most acute conditions. Hence, we cannot generalise our findings to the most acute circumstances typically treated in acute wards.

Our outcome measure assesses the patient’s perspective with an instrument sensitive to experiences of distress and wellbeing. Although central, there may be important aspects of treatment outcomes not captured by this instrument, or it may not capture all types of distress equally well. Investigations from different angles could further validate or nuance the picture.

## Conclusions

There were no consistent significant differences in improvement between the two regimens, and our analyses suggest that the improvement in MFT is noninferior to TAU. Importantly, participants in both regimens improved substantially. This indicates that supporting people in a less medication-oriented recovery process is not associated with any alarming reduction in treatment outcomes in the population currently receiving MFT. This is valuable information for people when choosing treatment services and may help reassure healthcare providers, clinicians, and other healthcare jurisdictions when considering different options.

## Implications and Future Directions

Our results suggest that MFT units are not inferior in terms of improvements in patient-reported distress by treatment end. Together with similar lines of research within populations with psychosis and depression, this indicates that treatment paradigms with less medication focus are not severely inferior to TAU (Breedvelt et al., 2021; Cooper et al., 2020; Cuijpers et al., 2020, 2023; Kappelmann et al., 2020; Leichsenring et al., 2022; Lichtenberg, 2011). Since shared decision-making about medications can be problematic in standard healthcare (Bjornestad et al., 2020; Blindheim, 2020; Haugom et al., 2020, 2022; Standal et al., 2021) it is important that such services remain an option. Future studies should focus on long-term follow-up results, outcomes for subgroups of patients and continue studying the status of shared decision-making in mental health care.

## Supplemental Material

sj-docx-1-isp-10.1177_00207640251390930 – Supplemental material for Are People Worse Off in a Mental Health Treatment Paradigm Where Medication Is Deemphasised? A Naturalistic Noninferiority Trial of an Initiative to Improve Patient Choice Supplemental material, sj-docx-1-isp-10.1177_00207640251390930 for Are People Worse Off in a Mental Health Treatment Paradigm Where Medication Is Deemphasised? A Naturalistic Noninferiority Trial of an Initiative to Improve Patient Choice by Kari Standal, Ole André Solbakken, Jūratė Šaltytė Benth, Allan Abbass and Kristin S. Heiervang in International Journal of Social Psychiatry
