# Supplementary material for: Are People Worse Off in a Mental Health Treatment Paradigm Where Medication Is Deemphasised? A Naturalistic Noninferiority Trial of an Initiative to Improve Patient Choice
Source: Int J Soc Psychiatry. 2025 Nov 23;72(4):981–96. doi: 10.1177/00207640251390930 (PMC13263461; doi:10.1177/00207640251390930)
Supplement: sj-docx-1-isp-10.1177_00207640251390930 – Supplemental material for Are People Worse Off in a Mental Health Treatment Paradigm Where Medication Is Deemphasised? A Naturalistic Noninferiority Trial of an Initiative to Improve Patient Choice [file sj-docx-1-isp-10.1177_00207640251390930.docx]

**Supplemental material**

Characteristics of the sample compared with those from other sources

Comparison of our research sample with all registered users included in hospital statistics during the recruitment period, where available, and compared with the quality project on OQ-45.

Table S 1 Gender, age, and treatment duration, compared with hospital statistics

| Regimen | Variable | Category | Statistic | **Research sample** | **All registered*** | **Difference (All – research sample)** |
| --- | --- | --- | --- | --- | --- | --- |
| MFT | Gender | Female | *n* (%) | 42 (71.2) | 60 (68.2) | 18 (–3.0) |
|  |  | Male | *n* (%) | 17 (28.8) | 28 (31.8) | 11 (3.0) |
|  | Age | | M (SD) | 38.6 (13.1) | 40.7 (13.3) | 2.1 (0.2) |
|  |  |  | Valid *n* | 59 | 88 | 29 |
|  | Treatment duration (weeks) | | M (SD) | 8.9 (2.2) | 7.6 (3.2) | –1,3 (1.0) |
|  |  |  | Valid *n* | 59 | 88 | 29 |
| TAU | Gender | Female | *n* (%) | 72 (58.1) | 273 (62.5) | 201 (4.4) |
|  |  | Male | *n* (%) | 52 (41.9) | 164 (37.5) | 112 (–4.4) |
|  | Age | | M (SD) | 43,7 (12.9) | 43.8 (13.4) | 0.1 (0.5) |
|  |  |  | Valid *n* | 124 | 437 | 313 |
|  | Treatment duration (weeks) | | M (SD) | 4.7 (2.2) | 3.1 (2.9) | –1.6 (0.7) |
|  |  |  | Valid *n* | 123 | 437 | 314 |

*Note*. *n* research sample = 183 (*n* MFT = 59, *n* TAU = 124), *n* all registered = 525 (*n* MFT = 88, *n* TAU = 437), MFT = Medication-free treatment, TAU = Treatment as usual.

**^a^** Hospital statistics including stays at the units during the recruitment period, excluding emergency admissions and readmissions within 30 days.

Table S 2 Main diagnoses at end of treatment compared with hospital statistics

| **Regimen** | **Diagnosis groups** | **Research sample** | | **All registered^a^** | | **Difference (All – research sample)** | |
| --- | --- | --- | --- | --- | --- | --- | --- |
|  |  | *n* | Valid % | *n* | Valid % | *n* | Valid % |
| MFT | Personality disorders F60–F61 | 12 | 20.3 | 14 | 16.1 | 2 | –4.2 |
|  | Psychosis F20–F29 | 9 | 15.3 | 13 | 14.9 | 4 | –0.3 |
|  | Bipolar disorder F30–F31 | 8 | 13.6 | 13 | 14.9 | 5 | 1.4 |
|  | Affective disorder. nonbipolar F32–F39 | 11 | 18.6 | 12 | 13.8 | 1 | –4.9 |
|  | Trauma/stress F43 | 6 | 10.2 | 10 | 11.5 | 4 | 1.3 |
|  | Anxiety F40–F41 | 5 | 8.5 | 8 | 9.2 | 3 | 0.7 |
|  | Dissociation F44 + F48.1 | 2 | 3.4 | 4 | 4.6 | 2 | 1.2 |
|  | Hyperkinetic disorder F90 | 4 | 6.8 | 4 | 4.6 | 0 | –2.2 |
|  | Obsessive compulsive disorder F42 | 0 | 0 | 3 | 3.4 | 3 | 3.4 |
|  | Drug-related disorders F10–F19 | 0 | 0 | 2 | 2.3 | 2 | 2.3 |
|  | Pervasive developmental disorders F84 | 1 | 1.7 | 2 | 2.3 | 1 | 0.6 |
|  | Somatoform disorders F45 | 1 | 1.7 | 1 | 1.1 | 0 | –0.5 |
|  | Eating disorders F50 | 0 | 0 | 1 | 1.1 | 1 | 1.1 |
|  | Valid | 59 |  | 87 |  | 28 |  |
| TAU | Affective disorder, nonbipolar F32–F39 | 37 | 30.3 | 92 | 22.5 | 55 | –7.8 |
|  | Psychosis F20–F29 | 12 | 9.8 | 82 | 20.0 | 70 | 10.2 |
|  | Personality disorders F60–F61 | 12 | 9.8 | 78 | 19.1 | 66 | 9.2 |
|  | Bipolar disorder F30–F31 | 20 | 16.4 | 53 | 13.0 | 33 | –3.4 |
|  | Trauma/stress F43 | 20 | 16.4 | 46 | 11.2 | 26 | –5.1 |
|  | Anxiety F40-41 | 9 | 7.4 | 17 | 4.2 | 8 | –3.2 |
|  | Pervasive developmental disorders F84 | 1 | 0.8 | 10 | 2.4 | 9 | 1.6 |
|  | Eating disorders F50 | 1 | 0.8 | 8 | 2.0 | 7 | 1.1 |
|  | Dissociation F44 + F48.1 | 0 | 0 | 7 | 1.7 | 7 | 1.7 |
|  | Drug-related disorders F10–F19 | 5 | 4.1 | 6 | 1.5 | 1 | –2.6 |
|  | Organic mental disorders F00–F09 | 1 | 0.8 | 2 | 0.5 | 1 | –0.3 |
|  | Obsessive compulsive disorder F42 | 1 | 0.8 | 2 | 0.5 | 1 | –0.3 |
|  | Somatoform disorders F45 | 0 | 0 | 1 | 0.2 | 1 | 0.2 |
|  | Other neurotic disorders F48 excluding F48.1 | 0 | 0 | 1 | 0.2 | 1 | 0.2 |
|  | Enduring personality change F62 | 1 | 0.8 | 1 | 0.2 | 0 | –0.6 |
|  | Mental retardation F70–79 | 1 | 0.8 | 1 | 0.2 | 0 | –0.6 |
|  | Hyperkinetic disorders F90 | 0 | 0 | 1 | 0.2 | 1 | 0.2 |
|  | Mixed disorders of conduct and emotions F92 | 0 | 0 | 1 | 0.2 | 1 | 0.2 |
|  | Other/unspecified developmental disorder F88–F89 | 1^b^ | 0.8 | 0 | 0 | –1 | –0.8 |
|  | Valid | 122 ^c^ |  | 409 |  | 287 |  |

*Note*. *n* research sample = 183 (*n* MFT = 59, *n* TAU = 124), *n* all registered= 525 (*n* MFT = 88, *n* TAU = 437), MFT = Medication-free treatment, TAU = Treatment as usual.

^a^Hospital statistics including stays at the units during the recruitment period, excluding emergency admissions and readmissions within 30 days.

^b^ Our research sample and hospital statistics were drawn from different sources (questionnaires and electronic journals). There may be errors or differences in registration, which may explain some inclusions in our research sample that were not included in the overall statistics.

^c^ One unknown: one patient received no F diagnosis.

Psychometrics

Table S 3 Cronbach’s alpha for multi-item scales

| Measure | Data set | Baseline | Weekly during treatment | End of treatment |
| --- | --- | --- | --- | --- |
| OQ-45-2 | Research sample | 0.923^a^ | 0.951^a^ | 0.956 ^a^ |
|  | Quality register | .941 | .948 | .955 |
| AII-42 | Research sample | 0.908 |  |  |

^a^ without missing values replaced

Missing replacements in research sample

Table S 4 OQ-45, missing items

|  | | Baseline | Weekly during treatment | Treatment end |
| --- | --- | --- | --- | --- |
| Items total | | 7833 | 29476 | 6085 |
| Items Missing | frequency | 87 | 584 | 125 |
|  | % | 1.1 | 2.0 | 2.1 |
| Little's MCAR test (*p*) | | 0.049 | .000 | .151 |

*Note*. MCAR = Missing completely at random

Examination of diagnosis subgroup: Diagnosis of either psychosis or bipolar disorder

Table S 5 Outcomes for participants with a diagnosis of either psychosis or bipolar disorder, research sample

| Outcome- measure | Regimen | *n* | M (SD) | *p* value | Cohen’s *d* (95% CI) |
| --- | --- | --- | --- | --- | --- |
| Change OQ-45 | MFT | 17 | 19.9 (23.1) | 0.351 | 0.3 (–0.3; 0.9) |
|  | TAU | 30 | 14.2 (17.8) |  |  |

*Note*. MFT = Medication-free treatment, TAU = Treatment as usual; Green cells = better score in the MFT group, Red cells = worse score in the MFT group

Table S 6 Baseline psychotropics

|  | | Range Less→More | Included | Source |  | | | | | | T-test | | | | |
| --- | --- | --- | --- | --- | --- | --- | --- | --- | --- | --- | --- | --- | --- | --- | --- |
|  |  |  |  |  | MFT | | | TAU | | |  |  | Cohen's d | | |
|  |  |  |  |  | M | SD | Valid | M | SD | Valid | t | p | Point estimate | 95% CI | |
| Adherence, mean all medicines pr user | | 1←4^b^ | U | P | 1,4 | 0,6 | 36 | 1,3 | 0,6 | 64 | 0,7 | 0,457 | 0,2 | -0,3 | 0,6 |
|  |  |  |  | C | 1,1 | 0,4 | 43 | 1,3 | 0,6 | 75 | -1,5 | 0,129 | -0,3 | -0,6 | 0,1 |
| Amount of use in standard doses^a^ | All psychotropics | | All | C | 1,9 | 1,7 | 59 | 2,3 | 1,8 | 124 | -1,5 | 0,135 | -0,2 | -0,5 | 0,1 |
|  | Antipsychotics | |  |  | 0,3 | 0,5 |  | 0,5 | 0,8 |  | -2,5 | **0,013** | -0,3 | -0,7 | 0,0 |
|  | Mood stabilizers (incl antiepileptics) | |  |  | 0,2 | 0,5 |  | 0,2 | 0,5 |  | 0,1 | 0,947 | 0,0 | -0,3 | 0,3 |
|  | Anxyliotics and hypnotics | |  |  | 0,5 | 0,9 |  | 0,5 | 0,7 |  | 0,0 | 0,974 | 0,0 | -0,3 | 0,3 |
|  | Antidepressants | |  |  | 0,7 | 1,2 |  | 0,8 | 1,1 |  | -0,6 | 0,576 | -0,1 | -0,4 | 0,2 |
|  | Medicine for hyperkinetic disorders and narcolepsia | |  |  | 0,1 | 0,3 |  | 0,0 | 0,2 |  | 0,8 | 0,445 | 0,1 | -0,2 | 0,4 |

*Note*. N=183 (n MFT= 59, n TAU=124), n Users of psychotropics=160 (n MFT=46, n TAU=114), MFT=Medication-free treatment, TAU=Treatment as usual, C= Clinician, P=Patient, U=Users of psychotropics, **Bold text**=significant at 0.05 level

^a^DDD= Defined daily dose according to WHO

^b^ Adherence:

1 Totally/mainly as prescribed

2 Mostly as prescribed

3 Partly as prescribed

4 Mainly not as prescribed

Table S 7 Time used medication at baseline

|  | | Antipsychotics | | Mood stabilizers | | Antidepressants | | Anxyliotics/  hypnotics | | Medicine for hyperactivity disorders and narcolepsia | |
| --- | --- | --- | --- | --- | --- | --- | --- | --- | --- | --- | --- |
| All | N | 54 | | 8 | | 43 | | 35 | | 1 | |
|  |  | Days | Years | Days | Years | Days | Years | Days | Years | Days | Years |
|  | M | 781,6 | 2,14 | 2213,9 | 6,07 | 651,0 | 1,78 | 1112,2 | 3,05 | 1936,0 | 5,30 |
|  | Mdn | 74,5 | 0,20 | 923,5 | 2,53 | 180,0 | 0,49 | 71,0 | 0,19 | 1936,0 | 5,30 |
|  | SD | 1486,2 | 4,07 | 2539,9 | 6,96 | 1264,4 | 3,46 | 2010,9 | 5,51 | 0 | 0 |
|  | Min | 7,0 | 0,02 | 31,0 | 0,08 | 11,0 | 0,03 | 14,0 | 0,04 | 1936,0 | 5,30 |
|  | Max | 5708,0 | 15,64 | 6126,0 | 16,78 | 6970,0 | 19,10 | 7931,0 | 21,73 | 1936,0 | 5,30 |
| MFT | N | 14 | | 4 | | 16 | | 14 | | 1 | |
|  |  | Days | Years | Days | Years | Days | Years | Days | Years | Days | Years |
|  | M | 1603,5 | 4,39 | 3884,3 | 10,64 | 1129,1 | 3,09 | 2164,9 | 5,93 | 1936,0 | 5,30 |
|  | Mdn | 568,0 | 1,56 | 4621,0 | 12,66 | 229,0 | 0,63 | 721,0 | 1,98 | 1936,0 | 5,30 |
|  | SD | 2004,4 | 5,49 | 2693,5 | 7,38 | 1919,6 | 5,26 | 2768,0 | 7,58 | 0 | 0 |
|  | Min | 19,0 | 0,05 | 169,0 | 0,46 | 25,0 | 0,07 | 39,0 | 0,11 | 1936,0 | 5,30 |
|  | Max | 5708,0 | 15,64 | 6126,0 | 16,78 | 6970,0 | 19,10 | 7931,0 | 21,73 | 1936,0 | 5,30 |
| TAU | N | 40 | | 4 | | 27 | | 21 | | 0 | |
|  |  | Days | Years | Days | Years | Days | Years | Days | Years | Days | Years |
|  | M | 494,0 | 1,35 | 543,5 | 1,49 | 367,7 | 1,01 | 410,4 | 1,12 |  |  |
|  | Mdn | 56,0 | 0,15 | 370,5 | 1,02 | 98,0 | 0,27 | 45,0 | 0,12 |  |  |
|  | SD | 1155,5 | 3,17 | 597,4 | 1,64 | 482,3 | 1,32 | 775,5 | 2,12 |  |  |
|  | Min | 7,0 | 0,02 | 31,0 | 0,08 | 11,0 | 0,03 | 14,00 | 0,04 |  |  |
|  | Max | 5533,0 | 15,16 | 1402,0 | 3,84 | 1402,0 | 3,84 | 2427,0 | 6,65 |  |  |

*Note*: Max duration per medication group per person

Table S 8 Frequency of medication use below and above half a year and 6 weeks at baseline

| Medication groups | Half year, cut point | MFT | | TAU | | 6 weeks, cut point | MFT | | TAU | |
| --- | --- | --- | --- | --- | --- | --- | --- | --- | --- | --- |
|  |  | n | % | n | % |  | n | % | n | % |
| Antipsychotics | <=182 days | 3 | 21,4 | 29 | 72,5 | <=42 days | 1 | 7,1 | 14 | 35,0 |
|  | >182 days | 11 | 78,6 | 11 | 27,5 | >42 days | 13 | 92,9 | 26 | 65,0 |
| Mood stabilizers | <=182 days | 1 | 25,0 | 1 | 25,0 | <=42 days | 0 |  | 1 | 25,0 |
|  | >182 days | 3 | 75,0 | 3 | 75,0 | >42 days | 4 | 100,0 | 3 | 75,0 |
| Antidepressants | <=182 days | 5 | 31,3 | 17 | 63,0 | <=42 days | 1 | 6,3 | 5 | 18,5 |
|  | >182 days | 11 | 68,8 | 10 | 37,0 | >42 days | 15 | 93,8 | 22 | 81,5 |
| Anxyliotics/hypnotics | <=182 days | 3 | 21,4 | 17 | 81,0 | <=42 days | 1 | 7,1 | 10 | 47,6 |
|  | >182 days | 11 | 78,6 | 4 | 19,0 | >42 days | 13 | 92,9 | 11 | 52,4 |
| Medications for hyperactivity/narcolepsia | <=182 days | 0 |  | 0 |  | <=42 days | 0 |  | 0 |  |
|  | >182 days | 1 | 100,0 | 0 |  | >42 days | 1 | 100,0 | 0 |  |

*Note*: Max duration per medication group per person

Questions from questionnaires used in this study that are not standardized instruments, translated from Norwegian

Information in gray is not used in the current article.

Self report at baseline

General introduction to the entire questionnaire:

**Questionnaire at beginning of treatment about you and your treatment in the last 6 months**

The questions are about background information, what is important for you, and treatment received in the last 6 months (before your current admission). Please check the best answer for each question. **Please give only one answer per question, unless otherwise specified.**

Regarding questions about your therapist or service provider, think of those involved in treatment of your mental health in the period.

This form is used only for this research and your therapist will not see your answers.

Birth year: _______

Gender

1 Male  2 Female  3 Other

General ending to the entire questionnaire:

Please check that you have answered all questions.

Thank you for giving us important information!

Clinician report at baseline and treatment end

General introduction to the entire questionnaire:

**Questionnaire for clinician at treatment start/end**

The form is filled out based on all the information one has and gets from the patient. Please fill in the top columns of each page in case they are separated.

| **Use of medication by mental disorders (indication)*** |
| --- |

*At treatment end the heading is **Use of medication by mental disorders (indication) *during admission***

The patient uses no medication

**The patient uses medication (fill in below about all current medications)**

**Depot medication for injection: ______________________** Dose: ______ mg Interval: _____ days Whether use of depot medication is voluntary or involuntary**:**  Voluntary  Coerced medication

**Current prescribed medications, daily dose, and scoring of side effects and adherence**

| **Medication**  (write clearly with capital letters) | **Medication group:**  1 Antidepressants  2 Antipsychotics  3 Tranquilizing/sleep  4 Mood stabilizing  5 Stimulants  6 Other  7 Don’t know | | **Daily dose (mg)** | **Weeks with this medication last 6 months** | **Used since**  **Month/year** | **Side effects**  1 None  2 Light  3 Moderate  4 Serious  5 Unknown | **Adherence**  1 Totally/mainly as prescribed  2 Mostly as prescribed  3 Partly as prescribed  4 Mainly not as prescribed  5 Unknown |
| --- | --- | --- | --- | --- | --- | --- | --- |
|  | |  |  |  |  |  |  |
|  | |  |  |  |  |  |  |
|  | |  |  |  |  |  |  |
|  | |  |  |  |  |  |  |
|  | |  |  |  |  |  |  |
|  | |  |  |  |  |  |  |
|  | |  |  |  |  |  |  |
|  | |  |  |  |  |  |  |

| **Duration of admission** | **Number of weeks** |
| --- | --- |
|  |  |

| **Current diagnoses** |
| --- |

**Main diagnosis ICD-10**

| **Diagnosis** |  | **Set year / month** | **How*** |
| --- | --- | --- | --- |
| F. |  |  |  |

**Other psychiatric diagnoses ICD-10 (also drug related diagnoses)**

| **Diagnosis** | **Set year / month** | **How*** |
| --- | --- | --- |
| F. |  |  |
| F . |  |  |
| F. |  |  |
| F . |  |  |
| F. |  |  |

***) Coding of “How” in tables above**

1 MINI/MINI-PLUSS

2 SCID-I

3 SCID-II

4 Other diagnostic tool:______________

5 Clinical consensus by two or more

6 Clinical evaluation by one

7 Don’t know
